# Orally bioavailable CB_2_ receptor agonists attenuate neuroinflammation and stabilize the blood–brain barrier

**DOI:** 10.21203/rs.3.rs-9705876/v1

**Published:** 2026-06-03

**Authors:** Slava Rom, Viviana Zuluaga-Ramirez, Sachin Gaghate, Uwe Grether, Wolfgang Guba, Nancy L. Reichenbach, Mario Van der Stelt, Christoph Ullmer, Pal Pacher, Yuri Persidsky

**Affiliations:** Temple University; Temple University; Temple University; Roche (Switzerland); Roche (Switzerland); Temple University; Leiden University; Roche (Switzerland); National Institute on Alcohol Abuse and Alcoholism; Temple University

**Keywords:** CB2 receptor agonists, BBB, neuroinflammation, leukocyte–endothelial interactions

## Abstract

**Background:**

Neuroinflammation and blood–brain barrier (BBB) dysfunction are key contributors to the pathogenesis of many neurological disorders. Cannabinoid receptor 2 (CB_2_) agonists possess anti-inflammatory properties and may represent promising therapeutic agents for preserving BBB integrity. This study evaluated the effects of novel orally bioavailable CB_2_ receptor agonists on leukocyte–endothelial interactions and BBB function using *in vitro* and *in vivo* models of neuroinflammation.

**Methods:**

Novel CB_2_ receptor agonists were tested in an aseptic localized encephalitis mouse model and in a lipopolysaccharide (LPS)-induced systemic inflammatory response model. Leukocyte adhesion and migration within cerebral microvessels were assessed *in vivo*. BBB permeability was evaluated in LPS-treated mice, while barrier integrity was measured in primary human brain microvascular endothelial cells (BMVECs) using transendothelial electrical resistance (TEER). Cytokine release, adhesion molecule expression (VCAM-1 and ICAM-1), and inflammatory gene expression in isolated cerebral microvessels were also analyzed. Statistical significance was determined using comparative analyses between treatment and control groups.

**Results:**

Among the compounds tested, RO6839828 and HU-910 demonstrated the greatest efficacy in reducing leukocyte adhesion to and migration across the BBB. CB_2_ receptor agonists significantly attenuated BBB hyperpermeability in LPS-treated mice and improved endothelial barrier integrity *in vitro*, as reflected by increased TEER values. Treatment also reduced cytokine release and decreased expression of VCAM-1 and ICAM-1. In addition, CB_2_ agonists attenuated dysregulation of multiple inflammation- and endothelial injury-related genes in cerebral microvessels isolated from LPS-treated mice. Pre-treatment of either BMVECs or primary human monocytes reduced monocyte adhesion to and transmigration across endothelial monolayers *in vitro*.

**Conclusions:**

Novel orally bioavailable CB_2_ receptor agonists reduced neuroinflammation-associated BBB dysfunction and leukocyte trafficking in both *in vitro* and *in vivo* models. These findings support the therapeutic potential of CB_2_ receptor agonists for the treatment of neurological diseases associated with neuroinflammation and BBB disruption.

## Introduction

Since the discovery of the two canonical cannabinoid receptors, CB_1_ and CB_2_, in the 1990s, cannabinoid medicinal chemistry has attracted sustained interest.. Over the past decade, numerous new chemical series have been identified that bind to cannabinoid receptors.. Because CB_1_ receptor agonists are associated with psychoactive effects, substantial effort has been directed toward the design of CB_2_-selective agonists, which have been implicated in anti-inflammatory and tissue-protective responses[1, 2]. Cannabinoids have been shown to modulate immune responses during inflammatory processes, and their immunosuppressive effects have been investigated in a broad range of disease models, including multiple sclerosis, diabetes and its complication, septic shock, rheumatoid arthritis, liver and kidney inflammation/fibrosis and others[2–7]. Animal studies indicate that cannabinoids exert immunomodulatory effects through several mechanisms: i) suppression of pro-inflammatory cytokine and chemokine production and enhancement of anti-inflammatory cytokine responses; ii) induction of apoptosis; iii) inhibition of cell proliferation; and iv) stimulation of regulatory T-cell responses[2, 8]. Our group recently demonstrated that activation of CB_2_ receptor with selective agonists interferes with VLA-4 and LFA-1 conformational changes and actin cytoskeletal remodeling in leukocytes, thereby regulating their ability to engage the brain endothelium, migrate across the blood–brain barrier (BBB), and prevent BBB injury. Selective activation of CB_2_ receptor in endothelial cells also reduces cytokine release and downregulates the expression of the adhesion molecules VCAM-1 and ICAM-1, which are required for leukocyte adhesion[9–11]. Most of the prior generation CB_2_ receptor agonists are neither selective, specific or suitable for *in vivo* administration[12].

Several novel, selective, and orally bioavailable CB_2_ receptor agonists have recently been synthesized and characterized. These include RO6839828, HU-910, RO6871304 (referred to as RO828, HU910, and RO304, respectively), as well as LEI-101 and LEI-102 [13–24]. These compounds are selective CB_2_ receptor agonists at both mouse and human receptors and have demonstrated therapeutic efficacy *in vivo* in models of acute and chronic kidney injury, acute liver injury and chronic liver fibrosis, hepatic cardiomyopathy, uveitis, traumatic brain injury, and amyotrophic lateral sclerosis[13–24].

In the current study, we investigated effects of these CB_2_ receptor agonists on neuro-inflammatory responses using our established model of aseptic encephalitis[25, 26]. Agonists were administered using a non-forceful oral feeding technique. Mice treated with the novel agonists exhibited reduced cerebral leukocyte–endothelial interactions, diminished expression of pro-inflammatory and vascular injury-related genes, and improved BBB integrity. Studies using *in vitro* BBB models further confirmed these *in vivo* observations. Taken together, our results demonstrate the anti-inflammatory effects of novel orally bioavailable and selective CB_2_ receptor agonists in monocytes and endothelial cells, supporting their potential therapeutic application in chronic inflammatory disorders both within and outside the CNS.

## Materials and methods

### Cells and reagents

#### Cells

Primary brain microvascular endothelial cells (BMVEC) were provided by Michael Bernas and Dr. Marlys Witte (University of Arizona, Tucson, AZ) as detailed previously (Bernas et al., 2010). Briefly, BMVEC were isolated from vessels of normal temporal cortex tissue derived from brain resections to remove eleptogenic foci in adult patients[27]. BMVEC were maintained in collagen-coated tissue culture ware in DMEM/F-12 media (Life Technologies, Carlsbad, CA) supplemented with 10% heat-inactivated fetal bovine serum (FBS)(Life Technologies), endothelial cell growth supplement (ECGS, BD Bioscience, Franklin Lakes, NJ), heparin (1 mg/ml, Sigma/Aldrich, St. Louis, MO), amphotericin B (2.5 μg/ml, Life Technologies), penicillin (100 U/ml, Life Technologies) and streptomycin (100 μg/ml, Life Technologies) and were used through passage 5 [28, 29]. Prior to experimentation, BMVEC monolayers were placed in medium as described above, but without the supplements, ECGS and heparin.

Primary human monocytes, isolated by countercurrent centrifugal elutriation from HIV-1/hepatitis B seronegative donors, were obtained from the University of Nebraska Medical Center (Ramirez et al., 2008, Rom et al., 2012) and were used within 24 hr of isolation. Monocytes were maintained in DMEM containing 10% heat-inactivated FBS, penicillin (100 U/ml), streptomycin (100 ug/ml), and L-glutamine (2 mM, Life Technologies) and were used within 24 hr of isolation.

##### Reagents

The CB_2_R ligands used in this study were acquired from Roche (RO6871304, RO6839828 and HU-910) or Leiden University (LEI-101 and LEI-102).

##### Animals and IVM

All animal experiments were approved by the Temple University Institutional Animal Care and Use Committee and conducted in accordance with the Temple University guidelines, which are based on the National Institutes of Health (NIH) guide for care and use of laboratory animals and with the ARRIVE (Animal Research: Reporting *In Vivo* Experiments) guidelines (study design, housing and husbandry, experimental practices, statistical methods) (www.nc3rs.org.uk/arrive-guidelines).

Mice were trained to voluntary accept 5 different CB_2_ agonists (HU-910, RO 304, RO 828, LEI-101 and LEI-102) once a day at a dose of 10 mg/kg, for 14 days. The animals were gently held by their scruff and fed using a 10 μl pipette. Dosage was tailored according to individual weights with a maximum volume of 7 μl per mouse. IVM was done in 8-week-old male C57BL/6 mice acquired from the Jackson Laboratory (Bar Harbor, ME). IVM for *in vivo* leukocyte adhesion was executed in mice that underwent craniotomy, cranial window and cannula implantation [11, 25, 26, 30]. Mice were allowed to recover for 6 days between implantation of the cannula and IC injections. IVM for *in vivo* leukocyte adhesion was performed on animals with cranial windows [11, 25, 26, 30]. Prior to IVM, animals were IC injected with TNFα (0.5 μg/mouse) with the volume not exceeding of 5 μL injected into the mouse brain, as described [26]. The above mentioned dose was established in dose-response experiments [25, 26], to achieve stable leukocyte infiltration complemented by mild brain edema [literature acknowledged signs of meningitis [31]], but without any unfavorable effects such as: high fever, seizures, locomotor deficiency and/or death [25, 26]. The Vybrant^®^ DiI Cell-Labeling Solution (DiI)(Life Technologies, Carlsbad, CA) introduced intravenously (i.v.) in order to visualize leukocytes. Leukocyte adhesion was detected in brain vessels through the cranial window using a Stereo Discovery V20 epifluorescence microscope, (Carl Zeiss Microimaging Inc., Thornwood, NY) equipped with a AxioCam MR digital camera, as previously described [11, 25, 26, 30], two hours after TNFα-injection. Transmigrated leukocytes were enumerated in an area covering a distance of 10 μm from the pial and parenchymal vessel wall by IVM. The extravasation of the leukocytes was calculated and normalized with respect to the immediate perivascular area surrounding the microvessel (MV) (10 μm from the endothelium × 100 μm long along the vessel), as described[25, 26, 30].

##### In vivo permeability assay

To evaluate protective effects of new cannabinoid agonists on BBB integrity, mice treated with or without agonists, were intraperitoneally (i.p.) injected with LPS to induce systemic inflammatory response, and animals received CB_2_ agonists i.p. at doses 2 mg/kg or 5 mg/kg simultaneously with LPS administration. 24 hours later all animals were injected i.p. with 20 U heparin followed by an i.p. injection of 200 μl of 2% sodium-fluorescein (Na-F) in saline. A Synergy 2 plate reader (BioTek, Winooski, VT) was used to evaluate the Na-F brain content and computed using external standards and the data are expressed as amount of tracer per mg of tissue, as described[25, 32, 33].

#### Mouse brain MV isolation and qRT-PCR arrays

Mouse brain MVs were isolated using a protocol based on previously published studies [25, 30, 32–35]. For every preparation, mice were overdosed with 5% isoflurane and their brains were harvested and placed in 4°C HBSS. The cerebellum, meninges, choroid plexus, brain stem, and large superficial blood vessels were removed. The remaining tissue was diced in HBSS (4 ml/gram) and then homogenized using a Potter-Thomas homogenizer (0.25mm clearance) (Thomas Scientific, Swedesboro, NJ). The homogenate was centrifuged (1000xg for 10 min at 4°C) to remove HBSS, re-suspended in 17.5% dextran (Sigma/Aldrich) and centrifuged again to separate the MVs. The MV pellet was re-suspended in 1% BSA in HBSS and the supernatant was centrifuged (4000xg for 10 min at 4°C). The MVs from each centrifugation were combined. The MV suspension was passed through a 100-*μ*m nylon mesh filter and then through a 40-*μ*m nylon mesh filter (Corning Life Sciences, Tewksbury, MA). The material retained on the 40-*μ*m nylon mesh filter contained the MVs.

RNA was isolated using the mirVana miRNA extraction kit (Life Technologies) and a PCR-based microarray assay (endothelial cell biology RT^2^-profiler PCR Array (PAMM-015Z) (SA Biosciences Corp., Frederick, MD)[25]) was utilized for evaluating the expression of genes involved in inflammatory responses and endothelial functions. Total RNA was converted to cDNA using RT^2^ PreAMP cDNA Synthesis Kit (Qiagen, Hilden, Germany). Data were examined using a web-based analysis tool (https://www.qiagen.com/us/shop/genes-and-pathways/data-analysis-center-overview-page/?akamai-feo=off) (SA Biosciences/Qiagen). Specific primers and probes for claudin-5, occludin, MMP-9, ICAM-1 and VCAM-1 genes were obtained from ThermoFisher Scientific and analyses were executed using the StepOnePlus real-time PCR system (ThermoFisher Scientific). Amplification was examined using the ΔΔCt method, using above mentioned web-based data investigation tool (SABiosciences/Qiagen) by normalization to housekeeping genes and fold-change calculated from the difference between experimental condition and untreated control. Fold changes of < 2 or > 2 were considered significant. Data are presented from two independent experiments, and each group is comprised of 3–4 individual mice. To exclude data bias, two people performed analysis in blinded fashion.

##### Monocyte adhesion assays.

Quantitative adhesion assays were performed as previously described [29]. BMVEC were seeded on collagen type I coated 96-well black wall clear bottom plates (Corning Costar Inc, Corning, NY) at a density of 2 × 10^4^ cells/well. Confluent BMVEC monolayers or monocytes were then treated with/without CB_2_ agonist as described in figures. All treatments were removed from the monocytes prior to labeling with the fluorescent tracer, calcein-AM (5 μM, Life Technologies). Labeled monocytes (1 × 10^5^ cells/well) were added to the BMVEC, incubated together for 15 min and rinsed 3 times with 1X PBS to eliminate non-adherent monocytes. The fluorescence of adherent monocytes was measured on a Synergy 2 plate reader (BioTek, Winooski, VT). The results for adhesion are represented as the mean ± SEM fold adhesion (number of adherent monocytes for each experimental condition divided by the basal adhesion of untreated control).

#### Transendothelial migration assays

Transendothelial migration assays were performed as previously described [29]. Briefly, BMVEC were seeded at 2 × 10^4^ cells/well on collagen-coated Fluoroblok 24-well plates (3 μm pore, BD Bioscience) and maintained for 6 days prior to migration assay. BMVEC were pretreated with/without CB_2_ agonist for 1 hr and then stimulated with TNFα (20 ng/ml) for 16 hr. Treatments were removed prior to monocyte introduction. Monocytes were incubated with/without CB_2_ agonist for 16 hr. All treatments were removed from the BMVEC prior to addition of monocytes. Calcein-labeled monocytes (1 × 10^5^ cells/well) were added to the BMVEC monolayers and chemotaxis was allowed for 2 hr. MCP11–1/CCL2 (30 ng/mL, Peprotech) was used as a relevant chemokine. The number of migrated monocytes was determined using Fiji software (National Institutes of Health, Bethesda, MD). The data are shown as fold difference in migration (mean ± SEM) from triplicate determinations, calculated from the number of migrated monocytes for each experimental condition divided by the number of migrated monocytes in the untreated, no chemoattractant control.

#### Transendothelial Electrical Resistance (TEER)

To determine the integrity of brain endothelial monolayers, TEER measurements were performed using the 1600R ECIS system (Applied Biophysics, Troy, NY) as previously described (Ramirez et al., 2009; Rom et al. 2016). The ECIS system provides real-time monitoring of changes in TEER. In brief, BMVEC at 2×10^4^/well were plated on collagen type I coated 96W20idf electrode arrays (Applied Biophysics) and maintained until confluent monolayers formed with stable TEER values. After seven days (with media change every three days), the monolayers were treated with CB_2_ agonist as indicated in the figure. The readings were acquired continuously at 4000 Hz at 30 min intervals. The data is shown as the percent change from the baseline TEER along with the SEM from at least three independent experiments containing at least four condition replicates.

### Statistical Analysis

Multiple group comparisons were performed by one-way analysis of variance with Tukey posthoc tests (*in vivo* and *in vitro* adhesion and migration assays, permeability and qPCR). Statistical analyses were performed utilizing Prism v6.0c software (GraphPad Software, San Diego, CA). Data are expressed as the mean ± SEM of experiments conducted multiple times. Differences were considered significant at p < 0.05. Different people did assays and analyses in blinded manner to reduce bias.

## Results

### Novel CB _2_ agonists diminish leukocyte adhesion to and migration across the endothelium, and protect barrier function in vivo

In the current study, we investigated the effects of novel, structurally diverse, orally bioavailable, and selective CB2 receptor agonists on neuroinflammatory responses using our established model of aseptic encephalitis. These previously characterized agonists (phytocannabinoid derived HU-910, triazolopyrimidine class RO6871304, 2,4,5-trisubstituted pyrazine derived RO6839828, and imidazolidine-2,4-dione compound class LEI-101 and LEI-102) have demonstrated therapeutic benefit *in vivo* across diverse disease models, including acute and chronic renal injury, acute hepatic injury, chronic liver fibrosis, hepatic cardiomyopathy, uveitis, traumatic brain injury, and amyotrophic lateral sclerosis[15, 22].

**Figure 1:**
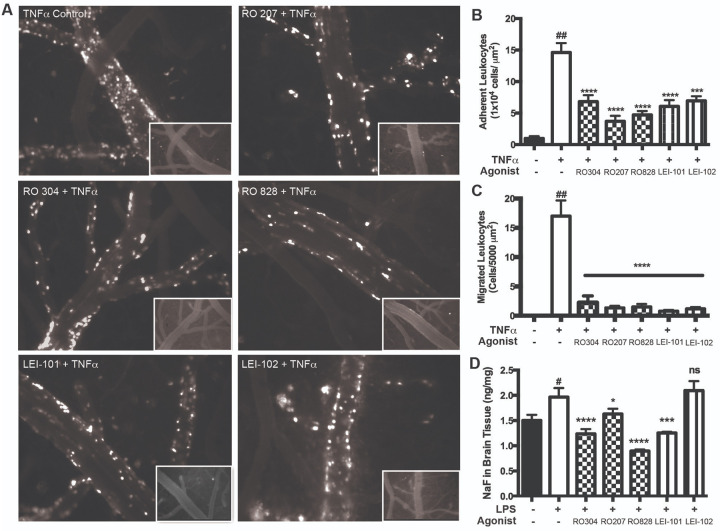
CB_2_ Agonists reduced leukocyte adhesion and migration in a model of aseptic encephalitis using a non-forceful feeding technique and improve BBB tightness. Mice were trained to voluntary accept 5 different CB_2_ agonists (HU-910, RO304, RO828, LEI-101 and LEI-102) as described in methods. Mice were intracerebrally injected with TNFa (0.5 ug/mouse) via cranula, and 2 h after insult, videos were taken using IMV in 3 different fields of view (from at least three animals per group). Representative images with baseline from the same vessel (inserts) from different conditions are shown (A). Quantitative measurements of leukocytes under firm adhesion (B) and migrated (C). Permeability was measured by 2% sodium fluorescein (NaF) tracer (D) in mice treated with or without LPS (5 mg/kg/IP) and pre-treated 2 hr prior to insult with CB_2_ agonists (from at least five animals per group). The results are shown as the mean adhesion ± SEM. (****) denotes a difference of *P* < 0.0001, (***) denoted a difference of *P* < 0.001, (*) denotes a difference of *P* < 0.05 between the CB_2_ agonist-treated animals and non-CB_2_ treated. (##) or (#) denote a difference of *P* < 0.01 or P<0.05 between non-CB_2_-treated and non-stimulated mice and non-CB2-treated and TNFa or LPS-stimulated, respectively.

We used a non-forceful oral administration protocol in which mice were trained to voluntarily consume five different CB2 agonists-RO207/HU-910, RO304, RO828, LEI-101, and LEI-102-once daily at a dose of 10 mg/kg.. Five days after placement of a cranial window with adjacent cannula, mice received an intracerebral injection of TNFα (0.5ug/mouse). Leukocyte–endothelial interactions and leukocyte migration across the cerebral endothelium were assessed 2 h after TNFα challenge by intravital microscopy.. Intracerebral administration of TNFα induced a 15-fold increase in leukocyte adhesion to the brain endothelium (Fig. 1A, B). Treatment with the CB_2_ agonists, RO304, LEI-101 and LEI-102 reduced leukocyte adhesion by 50–55%, whereas RO828 and HU-910 decreased it by 70 and 75%, respectively (Fig. 1A,B). TNFα challenge also led to a major increase in leukocyte migration across blood brain barrier in these animals (15–20 leukocytes per 5000 μm^2^, Fig. 1C). All CB_2_ agonists substantially reduced leukocyte migration by 89–94% (Fig. 1C).

Next, to evaluate whether novel CB_2_ agonists have any effect on BBB permeability under inflammatory conditions, we employed an animal model of systemic inflammation. In order to induce systemic inflammation leading to enhanced leukocyte adhesion to brain endothelium and increased BBB permeability[25], mice were injected ip with LPS (5 mg/kg). Animals were pretreated with CB_2_ agonists 2 h before LPS challenge, with five mice included in each treatment group. BBB permeability was measured by using 2% sodium fluorescein (NaF) tracer injected i.p. 30 min before tissue collection (200 μl/mouse), as previously described[11, 25, 30]. Quantitative analysis of NaF in brain tissue showed a 30% increase in BBB permeability in LPS-treated animals. Pretreatment with RO304, RO828, or LEI-101 completely prevented this LPS-induced increase in permeability, whereas RO207/HU-910 reduced it by 87%. In contrast, LEI-102 showed no protective effect at the tested dose (Fig. 1D), although it reduced permeability by 50% when administered at a higher dose (5 mg/kg; data not shown). Together, these findings indicate that the tested CB_2_ receptor agonists exert significant anti-inflammatory and barrier protective properties *in vivo*.

#### Novel CB_2_ agonists decrease inflammatory response and endothelial cell injury

**Figure 2:**
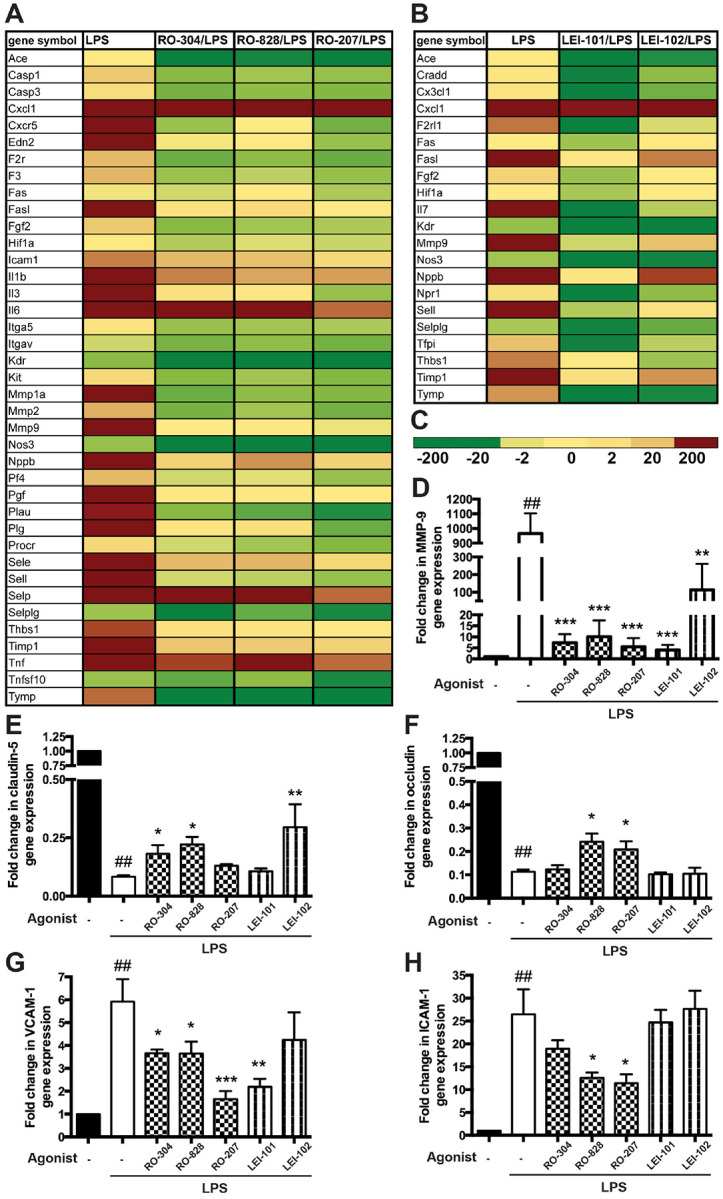
RO and LEI compounds diminished expression of inflammatory and barrier-compromising molecules in brain microvessels of mice with systemic inflammation (LPS injection). Total RNA was extracted from microvessels and a PCR-based microarray assay for evaluating the expression of genes was done as described in methods. Fold changes of <2 or >2 were considered significant. Data presented from triplicate determinations for HU-910 and RO (A) as well as LEI (B) compounds, if at least one of the compounds significantly (p<0.05) changed gene expression vs non-CB_2_ LPS-treated mice. C. Represents the scale of the gene regulations for A and B. qPCR data of MMP9 (D), claudin-5 (E), occludin (F), VCAM-1 (G) and ICAM-1 (H) genes. qPCR was done in triplicates for each mouse (n=3). The results are shown as the mean ± SEM. (*), (**) and (***) denote a difference of *P* < 0.05, P<0.01 and P<0.001 between the CB_2_ agonist-treated animals and non-CB_2_ treated. (##) denotes a difference of P<0.05 between non-CB2-treated and non-stimulated mice and non-CB_2_-treated and LPS-stimulated.

To identify inflammatory response–associated genes modulated by CB_2_ receptor activation in brain endothelial cells, we profiled the expression of genes involved in inflammation and autoimmunity regulation in microvessels isolated from LPS-challenged mice. Animals were exposed to LPS (5 mg/kg) and pretreated with the novel CB_2_ receptor agonists by oral administration, as described above. Using a commercial PCR-based array, 92 genes relevant to inflammatory and endothelial cell injury responses, cell adhesion molecules, angiogenesis were analyzed in microvessels isolated from untreated mice, LPS-exposed mice, and mice treated with LPS plus CB_2_ receptor agonist. The array analyses revealed that 71 gene targets were up-regulated and three down-regulated more than 2-fold by the LPS when compared to untreated controls. RO304, RO828 and HU-910 compounds reversed these changes in 46, 18 and 32 genes, respectively (p<0.05 (Fig. 2A), while LEI-101 and LEI-102 attenuated alterations in gene expression in 33 and 4 genes respectively (p < 0.05) (Fig. 2B). Notably, interleukins (IL-1β, IL-3, and IL-6 up-regulated by 1,000–47,000-fold) showed 90–99.5% attenuation in HU-910 and RO agonists treated mice, whereas LEI agonists did not have significant effect on these interleukin genes. The expression of adhesion molecules was markedly upregulated in microvessels from LPS-treated mice, with E-selectin, P-selectin, and L-selectin increasing by 250- to 1,500-fold. Treatment with HU-910, RO-series compounds, and LEI-series CB2 agonists reduced the expression of these adhesion molecules by 50–99.5%, although LEI-101 significantly reduced only L-selectin. ICAM expression was also strongly induced by LPS, increasing approximately 95-fold, and this response was attenuated by 50–80% only by HU-910 and RO-series compounds. To confirm array data, we performed qPCR on gene encoding adhesion molecules ICAM and VCAM. VCAM gene expression was up-regulated by LPS by around 6-fold which was reduced by 50–55% by RO304 and RO828 agonists, and by 70–75% by HU-910 and LEI-101 (Fig. 2G). Only RO828 and RO207 were able to significantly reduce ICAM expression in microvessels (Fig. 2H). Notably, expression of the tight junction protein claudin-5 was significantly downregulated in microvessels from LPS-treated mice, decreasing approximately 11-fold. Treatment with the CB_2_ receptor agonists RO304, RO828, and LEI-102 partially attenuated this downregulation, increasing claudin-5 expression by 2- to 3-fold relative to untreated LPS-exposed mice (Fig. 2E). Similarly, expression of another tight junction protein, occludin, was reduced approximately 10-fold under systemic inflammatory conditions. This decrease was partially reversed by RO828 and RO207/HU-910, which increased occludin expression by 2- to 2.4-fold compared with untreated LPS-exposed mice (Fig. 2F). These findings suggest that inflammation-induced reductions in tight junction protein expression may contribute to BBB leakiness, whereas CB_2_ receptor agonists may help preserve barrier integrity through systemic anti-inflammatory and barrier-protective effects. TNFα expression was markedly induced in LPS-treated animals, increasing approximately 1,100-fold, and was suppressed by 70–90% following treatment with HU-910 and RO-series CB2 agonists. Expression of matrix metalloproteinases implicated in BBB disruption, including MMP-1, MMP-2, and MMP-9, was also strongly upregulated in LPS-exposed mice, increasing by 57- to 9,400-fold. Treatment with CB2 agonists attenuated this induction by 80–99% (Fig. 2A, B, D).

### In vitro effects of novel CB _2_ agonists.

**Figure 3:**
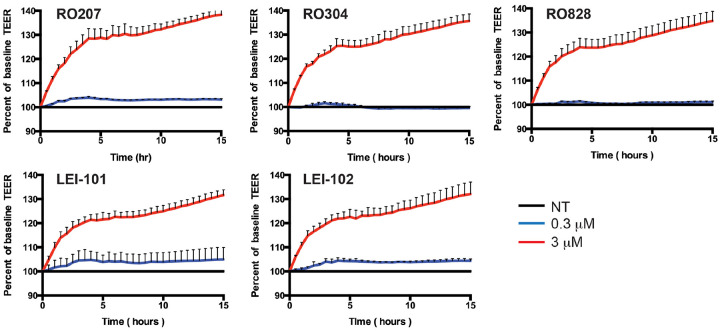
Novel CB_2_ agonist enhances barrier function in human BMVEC. TEER, an indicator of barrier integrity, was measured (by ECIS) in monolayers untreated or treated with the CB_2_ agonists, HU-910, RO304, RO828, LEI-101 or LEI-102 (0.3 or 3 μM). CB_2_ agonists induced tightening of the barrier (7–28%) in dose- and time-dependent manner. TEER was performed on primary BMVECs from 2 donors in quadruplicates. The results are shown as the mean ± SEM.

**Figure 4:**
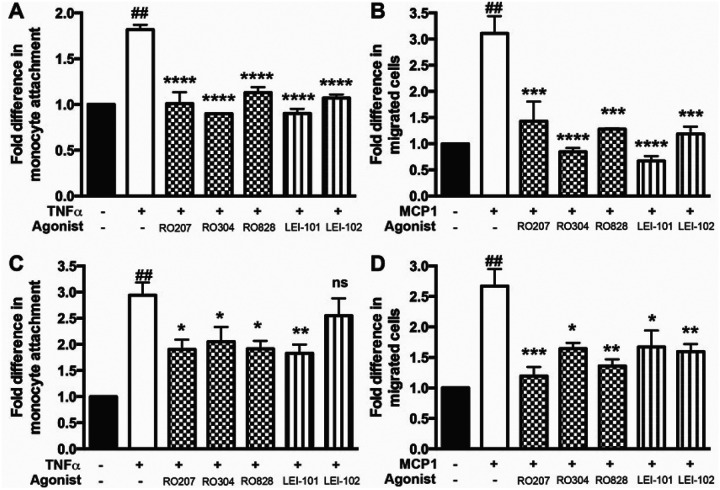
Treatment of either monocytes (A,B) or BMVEC (C,D) with novel CB_2_ agonist attenuates monocyte adhesion to (A,C) and migration across (B,D) BBB models. Monocytes were cultured separately for 16 hr in the absence or presence of CB_2_ agonist at 0.3 μM (for adhesion assay) (A) or 3 μM (for migration assay) (B); treatments were removed prior to addition of monocytes to BMVEC monolayers, that were treated for 16 hr with TNFα (20 ng/ml). BMVEC were pretreated for 1 hr with or without CB_2_ agonist (0.3 μM) (C) or 3 μM (for migration assay) (D) and then exposed to TNFα (20 ng/ml) for 16 hr; treatments were removed prior to monocyte addition. Data are presented as fold difference (mean ± SEM) of adhesion compared to basal adhesion in untreated cells (*p<0.05 vs. untreated control). For migration, Calcein-AM labeled monocytes were added to the upper chamber of FluoroBlok inserts and MCP1 (30 ng/mL) was used as a relevant chemokine. Chemotaxis was allowed for 2 hr. Data are shown as means of at least triplicate determinations ± SEM. (*), (**), (***) and (****) denote a difference of *P* < 0.05, P<0.01, P<0.001 and P<0.0001 between the CB_2_ agonist-treated cells and non-CB_2_ treated. (##) denotes a difference of P<0.05 between non-CB_2_-treated and non-stimulated mice and non-CB_2_-treated and TNFα –stimulated in adhesion or TNFα and with MCP1–stimulated cells for migration assay.

We first evaluated the cytotoxicity of all five CB_2_ receptor agonists (RO828, RO304, HU-910, LEI-101, LEI-102) in primary human BMVEC. Reduced cell viability was observed at concentrations only of 50 μM or higher for all compounds tested (data not shown). We next examined whether CB2 activation enhances barrier integrity under physiological conditions. Treatment with the highly selective novel CB_2_ receptor agonists increased the tightness of BMVEC monolayers in a dose- and time-dependent manner, producing a 27–38% increase over a 15 h period (Fig. 3). Improved barrier function was detectable as early as 0.5–2 h after agonist treatment. Next, we determined whether CB_2_ stimulation in human monocytes would decrease their adhesion to primary human BMVEC *in vitro*. TNFα activation of endothelium induced a 2.5-fold increase in the number of monocytes attaching to BMVEC. When monocytes were pretreated with novel CB_2_ agonists they reduced the adhesion of monocytes to BMVEC to levels seen in non-activated BMVEC (Fig. 4A). Using an *in vitro* BBB model, we examined whether CB_2_ receptor activation in primary human monocytes could inhibit their transmigration across BMVEC monolayers toward MCP-1/CCL2, a chemokine that is upregulated in the CNS under several neuroinflammatory conditions, including HIV-associated CNS infection and multiple sclerosis. [36]). Application of MCP-1 to the lower chamber of BBB constructs increased monocyte migration 3.1-fold. Pretreatment of monocytes with CB_2_ agonist attenuated their migration across endothelial monolayers by 80–100%, close or at levels seen in constructs without CCL2 application (Fig. 4B).

Next, we tested whether CB_2_ stimulation of BMVEC decreases monocyte adhesion to brain endothelium. BMVEC monolayers were activated with TNFα (mimicking neuroinflammatory conditions) in the presence of the novel CB_2_ agonists. Primary human monocytes were placed on the BMVEC only after all treatments were removed and the media changed. TNFα up-regulated adhesion 3-fold and four CB_2_ agonists diminished immune cell adhesion by ~ 50%, while pretreatment with LEI-102 had no effect (Fig. 4C). Using migration assays in an *in vitro* BBB model, we determined whether CB_2_ activation in endothelial cells could prevent monocyte passage across BMVEC monolayers using MCP-1 as a relevant cytokine. Application of MCP-1 to the lower chamber of BBB constructs increased monocyte migration 2.7-fold. Pre-treatment of BMVEC with all CB_2_ agonists attenuated monocyte migration across endothelial monolayers 65–89% (Fig. 4D). There was no difference in monocyte adhesion/migration in unstimulated BMVEC treated with CB_2_ agonists (data not shown).

## Discussion

In the current study, we combined complementary *in vivo* and *in vitro* approaches to demonstrate the anti-inflammatory potential of novel orally bioavailable CB_2_ receptor agonists. Utilizing non-forceful feeding technique and recently developed model of aseptic encephalitis [25, 26], we show that all five CB_2_ receptor agonists effectively reduced leukocyte adhesion to, and migration across, the BBB. We further demonstrate that the tested CB_2_ receptor agonists protected against BBB hyperpermeability in a model of LPS-induced systemic inflammation, a process associated with leukocyte engagement of the brain endothelium[2, 9, 11, 25, 28, 37Endothelial cells constitute the first line of defense against tissue injury. During inflammation, endothelial activation leads to the release of inflammatory mediators and increased expression of adhesion molecules. Chemokine-recruited inflammatory cells adhere to the activated endothelium and release pro-inflammatory mediators and reactive oxidants, thereby promoting endothelial dysfunction and barrier disruption. Once the endothelium is damaged and inflamed, leukocytes can transmigrate into the CNS and contribute to neuronal and glial injury{Batkai, 2007 #1982, 38–42].

We profiled gene expression in microvessels isolated from CB_2_ receptor agonists-treated mice exposed to systemic LPS-induced inflammation. Of the 92 inflammation- and/or endothelial injury–related genes analyzed, 71 were dysregulated in microvessels from LPS-treated mice compared with non-LPS-treated controls. Among the CB_2_ receptor agonists tested, RO304, HU-910, and LEI-101 showed the greatest efficacy in reversing LPS-induced gene dysregulation, whereas RO828 and LEI-102 exhibited more modest protective effects. LPS is known to downregulate tight junction proteins, including occludin and claudin-5, thereby compromising BBB integrity [43, 44]. Treatment with the CB_2_ receptor agonists RO304, RO828, RO207/HU-910, and LEI-102 partially rescued this tight junction protein dysregulation, consistent with the observed improvements in BBB permeability. (Fig. 1). Novel CB_2_ receptor agonists effectively attenuated LPS-induced overexpression of the adhesion molecules VCAM-1 and ICAM-1[9, 37–41, 45]. This attenuation was functionally associated with dramatic decrease of leukocyte adhesion to, and migration across, the BBB in the aseptic encephalitis model (Fig. 1). Several mediators implicated in BBB injury and known to be upregulated during neuroinflammation or systemic inflammatory responses, including cytokines such as IL-1β, IL-6, and TNFα, as well as matrix metalloproteinases[28], were also efficiently suppressed by the novel CB_2_ receptor agonists.

Our *in vitro* experiments further confirmed the ability of the novel CB_2_ receptor agonists to prevent leukocyte adhesion to, and migration across, endothelial monolayers. Interestingly, higher agonist concentrations were required to inhibit leukocyte transmigration than adhesion, suggesting that these processes may be regulated through partially distinct mechanisms. Inhibition of adhesion is likely mediated by reduced endothelial activation and decreased expression of adhesion molecules [2, 9, 11, 46–48]. By contrast, leukocyte transmigration requires actin cytoskeletal remodeling and activation of small GTPases, which may account for the observed differences in dose responsiveness[2, 11, 25, 30, 42, 49–52]. The novel CB_2_ receptor agonists also enhanced barrier tightness, as demonstrated by a significant increase in TEER. This improvement in BBB integrity may reflect increased stability of tight junction proteins[44, 53–55], enhanced localization of these proteins at the plasma membrane[37] and/or stabilization of the actin cytoskeletal machinery and small GTPase signaling[49, 54–58]. Further studies are needed to delineate the specific effects of these new agonists on each of these processes.

Notably, the CB_2_ receptor agonists tested in this study differed in efficacy when compared side by side across the same *in vivo* models and *in vitro* assays. For example, although LEI-102 was comparable to the other compounds in suppressing leukocyte adhesion and transmigration *in vivo*, it was less effective in reducing MMP-9 expression and in reversing LPS-induced VCAM-1 and ICAM-1 upregulation. Overall, LEI-102 suppressed only four LPS-induced genes *in vivo*. In *in vitro* assays, LEI-102 exerted significant anti-inflammatory effects in most experimental settings, although it was less effective in the adhesion assay when BMVECs were pretreated. Nevertheless, all CB_2_ receptor agonists significantly increased the tightness of BMVEC monolayers, indicating a shared capacity to enhance endothelial barrier function.

These differences in efficacy may reflect compound-specific pharmacological and pharmacokinetic properties. Structurally distinct CB_2_ receptor agonists may differ in receptor affinity, intrinsic activity, biased signaling, metabolic stability, and tissue distribution. In the *in vivo* setting, differences in oral absorption, first-pass metabolism, plasma protein binding, brain and microvascular exposure, and local tissue concentrations could substantially influence efficacy. Physicochemical properties, including lipophilicity, aqueous solubility, and membrane partitioning, may also affect compound access to endothelial cells, circulating leukocytes, and perivascular compartments. Thus, a compound that is effective in preventing leukocyte trafficking may be less potent in regulating transcriptional responses if its cellular exposure, intracellular persistence, or signaling bias differs from that of other agonists. Conversely, the consistent improvement in TEER across all compounds suggests that CB_2_ receptor activation converges on barrier-stabilizing pathways, even when effects on inflammatory gene expression are more variable.

Our previous studies using commercially available CB_2_ receptor agonists demonstrated suppression of inflammatory genes in brain endothelial cells and macrophages, reduced leukocyte adhesion and migration *in vitro* and *in vivo*, and attenuation of BBB permeability[9, 11, 37]. However, those earlier compounds required intraperitoneal administration because of limited oral bioavailability and poor solubility. In contrast, the orally bioavailable agonists evaluated here provide a more translationally relevant platform for targeting CB_2_ receptor-dependent anti-inflammatory and barrier-protective pathways. In the present study, we demonstrated the efficacy and anti-inflammatory potential of novel orally available CB_2_ agonists across a range of *in vivo* and *in vitro* assays. Because CB_1_ receptor agonists are limited therapeutically by psychoactive effects at pharmacologically effective doses, the development of CB_2_-selective agonists devoid of psychoactive activity remains an important goal. This is especially relevant given the protective role of CB_2_ receptor activation in neuroinflammation, a process implicated in many neurodegenerative disorders.

## Supplementary Material

Supplementary Files

This is a list of supplementary files associated with this preprint. Click to download.

- SupplementalfiguresforCB2inflampaper20182026.doc
